# Inducible versus constitutive social immunity: examining effects of colony infection on glucose oxidase and defensin-1 production in honeybees

**DOI:** 10.1098/rsos.170224

**Published:** 2017-05-31

**Authors:** Margarita M. López-Uribe, Andrea Fitzgerald, Michael Simone-Finstrom

**Affiliations:** 1 Department of Entomology and Plant Pathology, North Carolina State University, Raleigh, NC 27695, USA; 2 Department of Public Health, University of North Carolina, Chapel Hill, NC 27599, USA; 3 Honey Bee Breeding, Genetics and Physiology Research Laboratory, USDA-ARS, Baton Rouge, LA 70820, USA

**Keywords:** *Apis mellifera*, qPCR, gene expression, American foulbrood

## Abstract

Honeybees use a variety of defence mechanisms to reduce disease infection and spread throughout the colony. Many of these defences rely on the collective action of multiple individuals to prevent, reduce or eradicate pathogens—often referred to as ‘social immunity’. Glucose oxidase (GOX) and some antimicrobial peptides (e.g. defensin-1 or Def1) are secreted by the hypopharyngeal gland of adult bees on larval food for their antiseptic properties. Because workers secrete these compounds to protect larvae, they have been used as ‘biomarkers’ for social immunity. The aim of this study was to investigate if GOX and Def1 are induced after pathogen exposure to determine whether its production by workers is the result of a collective effort to protect the brood and colony in response to a pathogen challenge. Specifically, we quantified GOX and Def1 in honeybee adults before and after colony-level bacterial infection by American foulbrood ((AFB), *Paenibacillus larvae*). Overall, our results indicate that levels of GOX and Def1 are not induced in response to pathogenic infections. We therefore conclude that GOX and Def1 are highly constitutive and co-opted as mechanisms of social immunity, and these factors should be considered when investigating immunity at the individual and colony level in social insects.

## 1. Introduction

**Figure 1.**
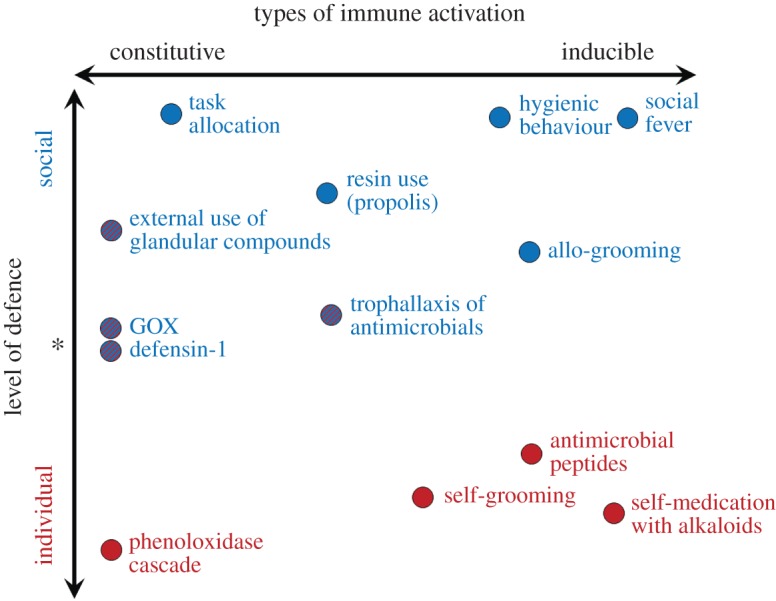
Framework for expression of social and individual immune traits ranging from highly constitutive to highly inducible. Social insects function, in many ways, as a ‘superorganism’. Both the social and individual immune systems have many analogous features and therefore language established for physiological immunity (constitutive versus inducible immunity) can be extended to the discussion of social immunity (see the electronic supplementary material, appendix 1 for more information on rationale and full citations for position of traits).

Social species rely on individual and group mechanisms to reduce the increased risk of disease transmission that results from living in large colonies [1,2]. When defence mechanisms rely on collective actions to prevent, reduce or eradicate diseases, they are referred to as ‘social immunity’ [1,3]. The honeybee (*Apis mellifera*) is a model organism to study the role of physiological and behavioural mechanisms on social immunity [4,5]. These defence mechanisms can result from individual or group defences and range on a spectrum from highly constitutive (regularly expressed as a first line of defence) to highly inducible (upregulated in response to exposure) (figure 1; [6]). For example, common inducible group behavioural defences of social immunity include allogrooming [7] and hygienic behaviours—defined as the ability of workers to remove diseased brood from the nest [8], while worker task allocation is highly constitutive [9]. In terms of individual physiological immunity, the phenoloxidase cascade is often described as a model for constitutive innate immunity [10,11], while antimicrobial peptides are highlighted as highly inducible, even though they show some constitutive expression [4].

One set of mechanisms of social immunity is based on the use of antimicrobial compounds to reduce the probability of pathogens entering the nest or spreading among individuals [12]. These antimicrobial compounds can be environmentally collected or self-produced. Honeybees collect plant resins to modify their nesting environments by inhibiting microbial growth with these externally gathered compounds [13–15]. In this case, collection of plant-produced resources is both constitutively expressed [14,16,17] and induced by pathogen exposure [18,19]. On the other hand, antimicrobial compounds produced by individuals can be externally secreted for group and food sanitation [20], as are venom gland products that are antiseptic and often found on cuticles and brood cells [21–23].

Two other self-produced compounds that are often considered biomarkers of social immunity—because of their roles in sanitizing honey stores and brood food—are glucose oxidase (GOX) and defensin-1 (Def1) [20,24,25]. GOX is an enzyme produced by the hypopharyngeal glands that converts β-d-glucose into d-gluconic acid and hydrogen peroxide (H_2_O_2_) through an oxidation reaction [26]. The antiseptic properties of H_2_O_2_ can prevent bacterial and fungal growth [26,27]. Even though levels of H_2_O_2_ production are positively correlated with the inhibition of pathogen development in larval food of honeybees (e.g. honey [28] and royal jelly [27]), it is unknown whether this antimicrobial secretion can be induced as a response to pathogen pressure. Def1 is an antimicrobial peptide, and unlike its other isoform defensin-2, it is also produced mainly by the hypopharyngeal glands of honeybees and found in the royal jelly fed to developing larvae [24].

In this study, we investigate whether GOX activity and Def1 expression are activated in nurse bees after a colony-level challenge with the causative agent of the bacterial brood disease American foulbrood ((AFB), *Paenibacillus larvae*). Specifically, we quantified levels of enzymatic activity of GOX and gene expression of GOX and Def1 for nurse bees in healthy and AFB-infected honeybee colonies to test whether or not these compounds are induced in nurse bees after colony-level exposure to pathogens. Honeybee larvae become infected with AFB by the transfer of spores via contaminated food, which come from spores left behind in re-used cells or from adult bees carrying them and then contaminating brood food [29]. Therefore, one hypothesis is that GOX and Def1 production is inducible in nurse bees because these compounds either; (i) directly increase in nurse bees owing to exposure to AFB spores leading to increased brood food sanitation and decontamination of the hive, or (ii) nurse bee physiology changes in response to the presence of infected larvae suggesting that these antimicrobial compounds are produced because nest-mates (larvae in this case) are sick. This change in physiology after nest-mate infection has recently been documented in queens [30]. The alternative hypothesis is that GOX and Def1 production are not inducible in either manner, meaning they are constitutively expressed and are co-opted as a social immune defence. Our results shed insights into the role of GOX and Def1 in the larger framework of the types of immune activation of individual and social immune system of honeybees and other social insects.

## 2. Material and methods

### 2.1. Experiment

We performed the experiment in Raleigh, NC, USA, using five frame nucleus colonies of *A. mellifera* for eight experimental colonies (five for AFB treatment and three controls), each containing three brood frames and one honey frame, a laying queen and equally sized adult bee populations. The queen of one of the control colonies died during the first week of the experiment, we therefore used two colonies as controls, and five colonies for the bacterial treatment using the AFB solution. Hives were located in an isolated area to prevent transmission of *P. larvae–*a.k.a. AFB–to healthy colonies*.* For bacterial infection treatment, we collected *ca* 30 AFB ‘scales’ or the dried remnants of symptomatic, dead larvae from an infected colony in Mocksville, NC, USA that was obtained from the North Carolina Department of Agriculture. Following standard protocols, scales were macerated with sterile deionized water, heat-shocked for 10 min at 80°C, and diluted in a 10% sucrose solution at a final concentration of 1.5 × 10^7^ spores ml^−1^. Three control colonies just received the sucrose solution. To infect the five treatment colonies, bees were shaken off each frame and combs were sprayed with a 5 ml solution on each side (following recipe in [31]). Before infection, we marked 100 newly emerged bees with coloured paint to identify age of bees. Fifteen age-marked individuals were collected at 7 and 14 days old before infection, and stored at −80°C. We inoculated treatment colonies with AFB during week 3, following the *T*_0_ 14 day old bee collection, and re-inoculated on week 4 (table 1). We repeated the infection procedure to guarantee that colonies would show AFB symptoms. The same marking and sampling procedures were repeated to collect 7 and 14 day old bees twice after infection (*T*_1_ and *T*_2_). AFB infections were quantified weekly throughout the experiment by counting the total number of symptomatic cells in the three brood frames for each colony.

**Table 1.** Summary of disease infection in experimental colonies through time. (Infection was quantified as the number of cells containing dead, symptomatic larvae or AFB ‘scale’ in colonies after initial infection.)

	week 1	week 2	week 3	week 4	week 5	week 6	week 7
AFB 1	0	0	0	0	26	91	228
AFB 2	0	0	0	5	67	89	71
AFB 3	0	0	0	3	6	178	30
AFB 4	0	0	0	1	58	57	125
AFB 5	0	0	0	15	143	342	^a^
control 1	0	0	0	0	0	0	0
control 3	0	0	0	0	0	0	0
bee marking	*T*_0_		*T*_1_		*T*_2_		
collection		7 d (*T*_0_)	14 d (*T*_0_)	7 d (*T*_1_)	14 d (*T*_1_)	7 d (*T*_2_)	14 d (*T*_2_)
treatment			AFB	AFB			

^a^Colony AFB 5 absconded before week 7.

### 2.2. Glucose oxidase enzymatic activity

We quantified the enzymatic activity of GOX from heads of 12 individuals per colony for each treatment and time period in our experiment. Head tissue was homogenized on ice in phosphate buffered saline (pH 7.4) using pestles. To quantify GOX activity, we used a peroxidase assay kit (ThermoFisher A22188) that produces the red compound resorufin with the presence of hydrogen peroxide (H_2_O_2_). In the presence of peroxidase, resorufin is produced in a 1 : 1 stoichiometry. Therefore, GOX activity was estimated as the maximum velocity (*V*_max_) of the rate of production of the red compound at an absorbance of 560 nm for 60 min. *V*_max_ was calculated as the slope of the linear phase of the reactions using the software KCJunior v. 1.22 (Bio-Tek, Winooski, VT, USA). Each reaction was repeated three times to estimate errors associated with sample preparation.

### 2.3. Gene expression

We quantified gene expression of *GOX* (F: GAG GGC GGA AAA TCA TCA GAC C; R: AGG ATT ACC CGA GAT CAC CTG C; [32]) and *Def1* (F: TGC GCT GCT AAC TGT CTC AG; R: AAT GGC ACT TAA CCG AAA CG; [33]) from the heads of eight individuals per colony for each treatment and time period using only the 14 day old bees. Briefly, total RNA was extracted using the Maxwell® system with the LEV simplyRNA tissue kit (Promega). cDNA synthesis was performed using the QuantiTect Reverse Transcription Kit (Qiagen Inc.) following the manufacturer's protocols. qPCR reactions were conducted using a CFX96™ Real-Time PCR (BioRad, Inc.). Amplification was performed in 10 µl volumes using PowerUP SYBR Green Master Mix (Applied Biosystems) under the following thermal protocol: 95°C hold for 20 s, 40 cycles of 95°C for 1 s, 60°C for 5 s followed by a melt-curve dissociation analysis. All reactions included three technical replicates and qPCR data were expressed as the threshold cycle (*C*_t_) values normalized to expression of *β-actin* (F: TGC CAA CAC TGT CCT TTC TG; R: AGA ATT GAC CCA CCA ATC CA; [34]) and calculated using the 2^−ΔΔCt^ method following standard protocols [35]. The average for the colony at *T*_0_ (pre-infection) was used as the calibrator, and so normalized expression values were made relative to this.

### 2.4. Statistical analysis

We estimated the average GOX enzymatic activity of the three technical replicates for enzymatic activity (GOX) and the expression data (*GOX* and *Def1*). Samples that showed standard deviations greater than 2 among replicates were inspected for outliers. We removed single values deviated more than ±2 from the other two replicates, or removed the whole sample if the two remaining replicates after outlier removal still deviated by more than 2. Generalized mixed linear models were built using ‘treatment’ and ‘time’ as fixed effects, and the variable ‘colony’ was included as a random effect. For the enzymatic GOX data, ‘plate’ was also included as a random effect to account for any variability from the specific groups of samples that was analysed in the same plate (31 samples and 1 negative control). Models for 7 and 14 day old bees were analysed separately. Linear models were analysed using the maximum-likelihood approach of the *lme* function of the R package nlme [36].

## 3. Results

**Figure 2.**
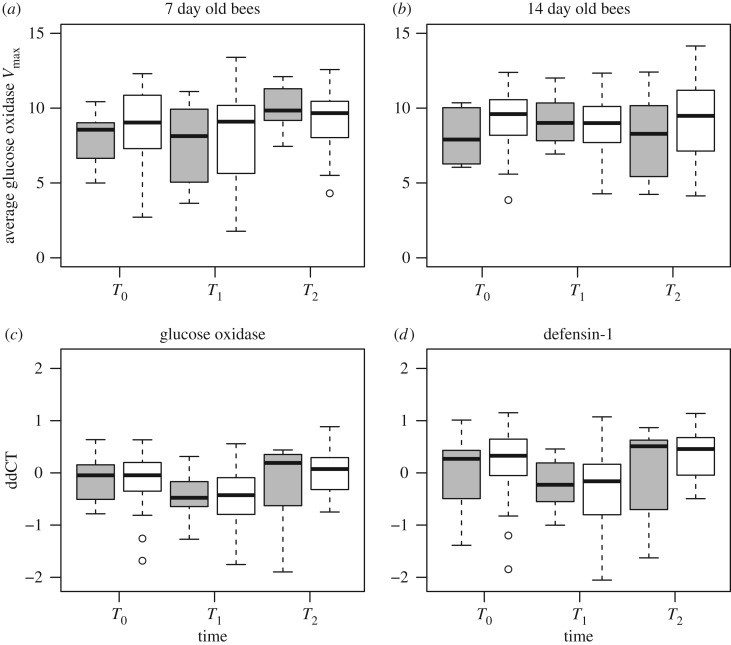
Levels of GOX enzymatic activity (*a*,*b*), and relative transcript abundances for *GOX* (*c*) and *Def1* (*d*) represented in boxplots. Each box represents the median (middle horizontal line), first and third quartile (upper and bottom horizontal lines, respectively), and the vertical lines extend to the maximum and minimums of the data, with the circles outside of these boundaries indicating outliers. Grey and white boxplots depict control and AFB treatment colonies, respectively.

The number of larval cells with overt AFB symptoms increased over time after initial infections in all experimental colonies, while control colonies showed no sign of diseased larvae (table 1). We did not detect a significant change in GOX activity between treatment and control colonies in 7 day (*F*_1,6_ = 0.01, *p* = 0.923) or 14 day old (*F*_1,5_ = 0.116, *p* = 0.752) bees (table 2; figure 2). For gene expression of *GOX*, there was no significant interaction between time and treatment (*F*_2,116_ = 1.385, *p* = 0.255) and no effect of AFB treatment (*F*_1,4_ = 0.007, *p* = 0.936) (figure 2). However, there was an overall significant effect of time (*F*_2,118_ = 8.672, *p* = 0.003), with bees at *T*_1_ having lower *GOX* expression than bees at *T*_0_ or *T*_2_. A similar finding was determined for *Def1*, with no significant effect for the interaction between time and treatment (*F*_2,116_ = 1.385, *p* = 0.255) or AFB treatment (*F*_1,4_ = 0.59, *p* = 0.485). There was an effect of time (*F*_2,116_ = 6.896, *p* = 0.015), with significantly lower *Def1* expression levels at *T*_1_ when compared with T_0_, and T_2_.

**Table 2.** Mixed linear models testing the effect of AFB treatment on levels of GOX enzymatic production for 7 and 14 day old bees, and transcript expression of *GOX* and *Def-1* in 14 day old bees. (*P*-values less than 0.05 (in italics) indicate a significant effect.)

	numDF	denDF	*F*-value	*P*-value
	GOX production—7 day old bees			
time	2	242	0.3979	0.6722
treatment	1	6	0.0103	0.9226
time × treatment	2	242	0.295	0.7451
	GOX production—14 day old bees			
time	2	181	0.0919	0.9122
treatment	1	5	0.116	0.7519
time × treatment	2	181	0.0164	0.9837
	*GOX* expression			
treatment	1	4	0.0072	0.9363
time	2	116	8.6718	*0.0003*
time × treatment	2	116	0.7687	0.4660
	*Def1* expression			
treatment	1	4	0.5901	0.4852
time	2	116	6.8964	*0.0015*
time × treatment	2	116	1.3848	0.2545

## 4. Discussion

GOX enzymatic activity and expression levels were not induced in 7 day old or 14 day old bees after honeybee colonies were challenged with a bacterial pathogen (AFB). Even though all infected colonies showed symptoms of disease, enzymatic production of GOX and transcription of *GOX* and *Def1* were unaffected at the colony level. The fact that *Def1* was not induced by exposure to AFB is not entirely unexpected. Even though Def1 is an antimicrobial peptide that responds to bacterial infection [37], AFB does not infect adults and only infects the larval stage. However, expression of antimicrobial peptides is typically used as a measure of inducible immunity for studies examining investment in mechanisms of individual immunity [10,11], even though they do show constitutive and genetically based expression patterns [38]. Our results therefore highlight the importance of considering whether these compounds can be induced in a social context. While there was no overall increase in expression after infection, we found a significant reduction in *GOX* and *Def1* expression levels during T_1_ for both control and treatment colonies. We are uncertain about the causes of this result and it is probably owing to various, wider environmental factors that were not measured in this experiment.

Our results indicate that even though GOX and Def1 are secreted into larval food for antiseptic properties, they remain constitutively expressed and are not increased owing to pathogen pressure. These findings are supported by previous work in GOX [24], and other types of constitutive immune defences such as metapleural [39] and venom gland secretions [21]. On the contrary to what we found in regard to these self-produced compounds, environmentally collected compounds, such as plant resins, can be induced after colony-level pathogen challenges [18,19]. Our data also suggest colony-level differences in GOX production, indicating that the genetic background of colonies may be a significant factor influencing the level of investment in expression of these antimicrobial compounds. In fact, one earlier study documented that colonies bred for resistance to AFB may have exhibited positive selection for constitutively increasing the level of antimicrobial compounds in the brood food [40], without knowing the responsible compounds. In this scenario, one mechanism of AFB resistance was found to be owing to an increase in antimicrobial activity of brood food, and thus those colonies displayed an increase in this particular mechanism as a constitutive social immune defence.

GOX activity and *Def1* expression have often been analysed as parameters of social immunity, as it has been hypothesized to prevent diseases through colony-food sterilization [41]. While the antiseptic role of these compounds is clear, it is unknown how colonies invest in these as mechanisms of social immunity. It is possible that the production of constitutive defences, such as GOX and Def1, may be maintained to prevent initial pathogen infection in social insects [40]. GOX has been detected in larval food of solitary bees [42], on grasshopper cuticles [43] and in other herbivorous insects [44], which suggests that the use of this enzyme as an antimicrobial secretion is an ancestral trait in non-social insects. As such GOX production in these different species fits the broader definitions of social immunity as proposed by Cotter & Kilner [2], and clarified by Meunier [3], whereby parental care and care of siblings can be described as an aspect of social immunity in social, communal and even solitary species.

Taken together, our results and the evidence of widespread use of GOX and Def1 suggest that some of the antimicrobial compounds produced by social insects can be co-opted to function as mechanisms of social immunity. Because social insects have stronger selective pressure of pathogen spread, they rely on a wide range of defences that include internal and external compounds, behavioural and physiological defences, constitutive or inducible mechanisms that benefit individuals and groups (figure 1; [4,6]). In addition, recent evidence in wood ants suggests that some species can mix antimicrobial compounds from self-produced and external sources, which increases their antimicrobial potential [45]. This highlights the need and ability of social insects to rely on both constitutive and inducible immune responses. The combination of the wide range of defences may therefore compensate for reduced physiological immune responses in social insects [46]. Understanding how colonies invest in defences across this continuum is key to gaining insight into the evolution of these defences and how pathogen pressure influences this investment.

## Supplementary Material

Expanded legend for Figure 1

## Data accessibility

Raw enzymatic activity and expression data are available as the electronic supplementary material: ‘Lopez-Uribe_RawData.xlsx’. We have submitted the raw data for the GOX enzymatic activity; and the GOX and Def1 expression data: http://datadryad.org/review?doi=doi:10.5061/dryad.0550m [47].
