# Supplementary material for: Inducible versus constitutive social immunity: examining effects of colony infection on glucose oxidase and defensin-1 production in honeybees
Source: R Soc Open Sci. 2017 May 31;4(5):170224. doi: 10.1098/rsos.170224 (PMC5451834; doi:10.1098/rsos.170224)
Supplement: Expanded legend for Figure 1 [file rsos170224supp1.docx]

**Supplementary Material – Appendix 1**

**
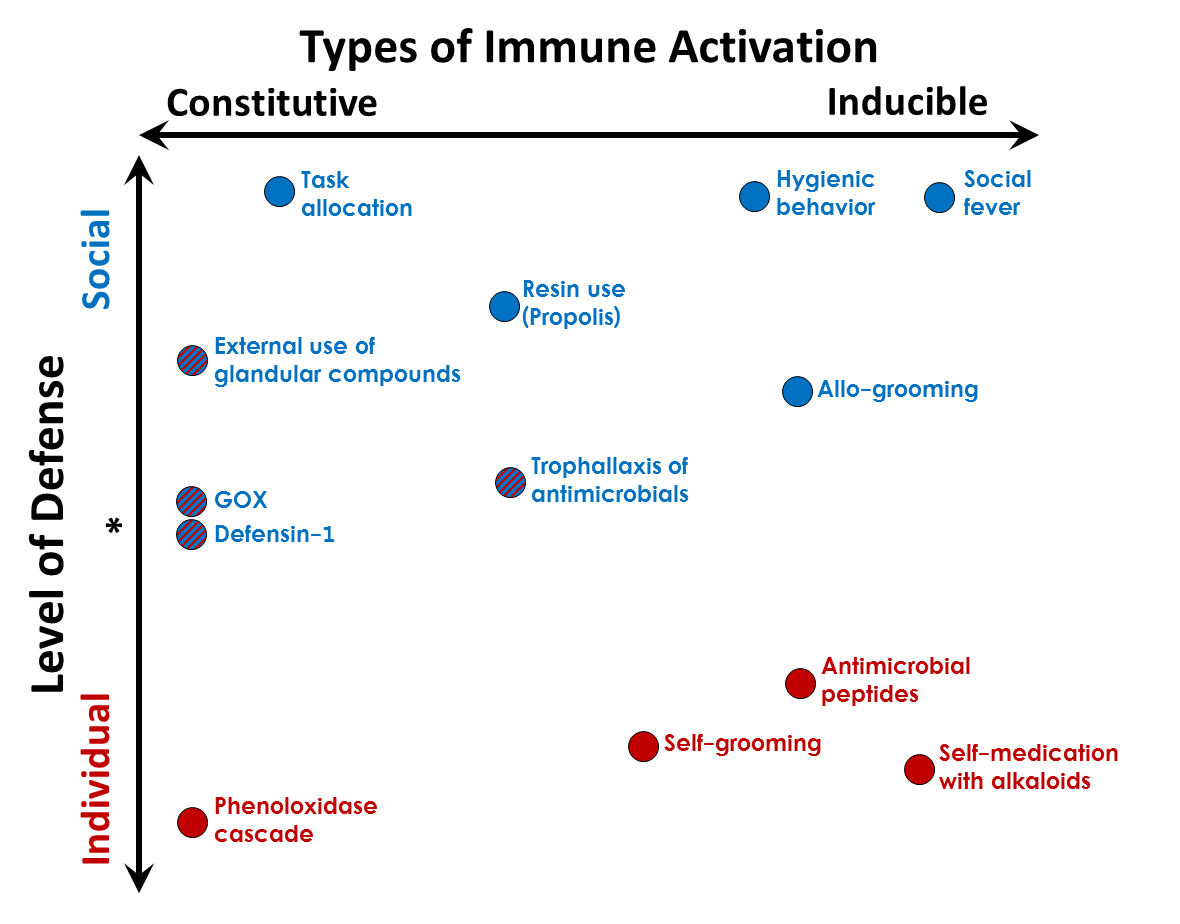
**

**Extended caption Figure 1.**

**Figure 1.** **Framework for discussion of the range of expression of social and individual immune traits ranging from highly constitutive to highly inducible.** Social insect colonies function, in many ways, as a “superorganism” (1-3), which is particularly relevant regarding parasite and pathogen defense. In this manner both the social and individual immune systems have many analogous features (4) and therefore language established for physiological immunity (constitutive vs. inducible immunity) can be extended to the discussion of social immunity (5). Selection of the location for each trait is based on the following, largely honey bee work, but other non-honey bee work was used for this framework as appropriate:

***1) Task allocation*** reduces exposure to and spread of disease, and only changes upon significant loss of one age or task group (6-8)

***2) External use of glandular secretions*** (e.g. venom and metapleural glands) is seen in wasps, bees and ants that spread self-produced compounds on their own cuticles, but also in some cases on others or on nest materials (9-14). These compounds may reduce both colony-level disease and individual infection

***3) GOX and Defensin-1*** in brood food and honey stores aligns more in the range of parental care, appears to be constitutive ((15-17), this paper)

***4)*** ***Resin use*** is largely constitutive but is also induced by certain pathogens in some species (18-25)

***5) Trophallaxis and exchange of antimicrobials*** may be increased in immune challenged individuals (26), but exchange of regurgitates has non-disease related benefits as well as potential benefits for social immunity (27, 28)

***6) Hygienic behavior*** is a highly inducible behavior. While individuals are constantly performing inspection-related behaviors to assess sick versus healthy larvae and pupae, the full suite of the behavior from detection to removal is induced by presence of disease and parasitism (29-32)

***7) Allogrooming*** of sick individuals has been seen to increase; allogrooming can lead to “social vaccination” of nestmates indicating an individual benefit to the behavior (33-35)

***8) Social fever***, an increase in brood/nest temperature, only occurs in response to challenge (36, 37)

***9) The phenoloxidase cascade*** is the major component of insect innate immunity, used to assess constitutive investment in immune function (38, 39)

***10) Self-grooming*** of parasites and pathogens can lead to decrease in individual infection, and at times, colony-level infection (34, 40, 41)

***11) Antimicrobial peptide*** expression is highly induced by infection, however they are regularly present at low levels in apparently healthy individuals (42). Use of some antimicrobial peptides seems to have been co-opted for social defenses and brood rearing, as in the case of Defensin-1 ((16, 17), this study)

***12) Self-medication with nectar antimicrobials and other environmental compounds*** occurs when pathogen-challenged bees selectively prefer certain nectar and nectar based antimicrobial compounds, some of which can reduce infections (43-46). This currently appears to be focused on the individual level, but could also have some social affects.

REFERENCES

1. Seeley TD. The honey bee colony as a superorganism. American Scientist. 1989;77(6):546-53.

2. Tautz J. The buzz about bees: biology of a superorganism: Springer Science & Business Media; 2008.

3. Wheeler WM. The ant-colony as an organism. Journal of Morphology. 1911;22(2):307-25.

4. Cremer S, Sixt M. Analogies in the evolution of individual and social immunity. Philosophical Transactions of the Royal Society B-Biological Sciences. 2009;364(1513):129-42.

5. Simone-Finstrom M. Social immunity and the superorganism: Behavioral defenses protecting honey bee colonies from pathogens and parasites. Bee World. 2017;94(01):21-29.

6. Naug D. Structure of the social network and its influence on transmission dynamics in a honeybee colony. Behavioral Ecology and Sociobiology. 2008;62(11):1719-25.

7. Naug D, Camazine S. The role of colony organization on pathogen transmission in social insects. Journal of Theoretical Biology. 2002;215(4):427-39.

8. Stroeymeyt N, Casillas-Pérez B, Cremer S. Organisational immunity in social insects. Current Opinion in Insect Science. 2014;5:1-15.

9. Baracchi D, Francese S, Turillazzi S. Beyond the antipredatory defence: Honey bee venom function as a component of social immunity. Toxicon. 2011;58(6-7):550-7.

10. Baracchi D, Mazza G, Turillazzi S. From individual to collective immunity: The role of the venom as antimicrobial agent in the Stenogastrinae wasp societies. Journal of Insect Physiology. 2012;58(1):188-93.

11. Tranter C, Graystock P, Shaw C, Lopes JFS, Hughes WOH. Sanitizing the fortress: protection of ant brood and nest material by worker antibiotics. Behavioral Ecology and Sociobiology. 2014;68(3):499-507.

12. Brütsch T, Jaffuel G, Vallat A, Turlings TCJ, Chapuisat M. Wood ants produce a potent antimicrobial agent by applying formic acid on tree-collected resin. Ecology and Evolution. 2017;7(7):2249-2254.

13. Graystock P, Hughes WOH. Disease resistance in a weaver ant, Polyrhachis dives, and the role of antibiotic-producing glands. Behavioral Ecology and Sociobiology. 2011;65(12):2319-2327.

14. Moreau SJM. “It stings a bit but it cleans well”: Venoms of Hymenoptera and their antimicrobial potential. Journal of Insect Physiology. 2013;59(2):186-204.

15. Alaux C, Ducloz F, Crauser D, Le Conte Y. Diet effects on honeybee immunocompetence. Biology Letters. 2010;6(4):562-565.

16. Klaudiny J, Bachanova K, Kohutova L, Dzurova M, Kopernicky J, Majtan J. Expression of larval jelly antimicrobial peptide defensin1 in Apis mellifera colonies. Biologia. 2012;67(1):200-211.

17. Vojvodic S, Johnson BR, Harpur BA, Kent CF, Zayed A, Anderson KE, et al. The transcriptomic and evolutionary signature of social interactions regulating honey bee caste development. Ecology and Evolution. 2015;5(21):4795-807.

18. Chapuisat M, Oppliger A, Magliano P, Christe P. Wood ants use resin to protect themselves against pathogens. Proceedings of the Royal Society B-Biological Sciences. 2007;274(1621):2013-2017.

19. Leonhardt SD, Bluthgen N. A Sticky Affair: Resin Collection by Bornean Stingless Bees. Biotropica. 2009;41(6):730-736.

20. Simone M, Evans JD, Spivak M. Resin collection and social immunity in honey bees. Evolution. 2009;63(11):3016-3022.

21. Simone-Finstrom M, Spivak M. Propolis and bee health: the natural history and significance of resin use by honey bees. Apidologie. 2010;41(3):295-311.

22. Castella G, Chapuisat M, Christe P. Prophylaxis with resin in wood ants. Animal Behaviour. 2008;75:1591-1596.

23. Castella G, Chapuisat M, Moret Y, Christe P. The presence of conifer resin decreases the use of the immune system in wood ants. Ecological Entomology. 2008;33(3):408-412.

24. Simone-Finstrom MD, Spivak M. Increased resin collection after parasite challenge: A case of self-medication in honey bees? Plos One. 2012;7(3):7.

25. Christe P, Oppliger A, Bancala F, Castella G, Chapuisat M. Evidence for collective medication in ants. Ecology Letters. 2003;6(1):19-22.

26. Hamilton C, Lejeune BT, Rosengaus RB. Trophallaxis and prophylaxis: social immunity in the carpenter ant *Camponotus pennsylvanicus*. Biology Letters. 2011;7(1):89-92.

27. Mirabito D, Rosengaus RB. A double-edged sword? The cost of proctodeal trophallaxis in termites. Insectes Sociaux. 2016;63(1):135-141.

28. Tragust S, Mitteregger B, Barone V, Konrad M, Ugelvig LV, Cremer S. Ants disinfect fungus-exposed brood by oral uptake and spread of their poison. Current Biology. 2013;23(1):76-82.

29. Wilson-Rich N, Spivak M, Fefferman NH, Starks PT. Genetic, Individual, and Group Facilitation of Disease Resistance in Insect Societies. Annual Review of Entomology. 2009;54:405-423.

30. Evans JD, Spivak M. Socialized medicine: Individual and communal disease barriers in honey bees. Journal of Invertebrate Pathology. 2010;103:S62-S72.

31. Spivak M, Reuter GS. Honey bee hygienic behavior. American Bee Journal. 1998;138(4):283-286.

32. Tragust S, Ugelvig LV, Chapuisat M, Heinze J, Cremer S. Pupal cocoons affect sanitary brood care and limit fungal infections in ant colonies. Bmc Evolutionary Biology. 2013;13: 225.

33. Konrad M, Vyleta ML, Theis FJ, Stock M, Tragust S, Klatt M, et al. Social transfer of pathogenic fungus promotes active immunisation in ant colonies. Plos Biology. 2012;10(4).

34. Theis FJ, Ugelvig LV, Marr C, Cremer S. Opposing effects of allogrooming on disease transmission in ant societies. Philosophical Transactions of the Royal Society B: Biological Sciences. 2015;370: 20140108.

35. Richard FJ, Aubert A, Grozinger CM. Modulation of social interactions by immune stimulation in honey bee, *Apis mellifera*, workers. BMC Biology. 2008;6.

36. Starks PT, Blackie CA, Seeley TD. Fever in honeybee colonies. Naturwissenschaften. 2000;87(5):229-231.

37. Simone-Finstrom M, Foo B, Tarpy DR, Starks PT. Impact of food availability, pathogen exposure, and genetic diversity on thermoregulation in honey bees (*Apis mellifera*). Journal of Insect Behavior. 2014;27(4):527-539.

38. Laughton AM, Boots M, Siva-Jothy MT. The ontogeny of immunity in the honey bee, Apis mellifera L. following an immune challenge. Journal of Insect Physiology. 2011;57(7):1023-1032.

39. Armitage SAO, Thompson JJW, Rolff J, Siva-Jothy MT. Examining costs of induced and constitutive immune investment in Tenebrio molitor. Journal of Evolutionary Biology. 2003;16(5):1038-1044.

40. Guzman-Novoa E, Emsen B, Unger P, Espinosa-Montaño LG, Petukhova T. Genotypic variability and relationships between mite infestation levels, mite damage, grooming intensity, and removal of Varroa destructor mites in selected strains of worker honey bees (Apis mellifera L.). Journal of Invertebrate Pathology. 2012;110(3):314-320.

41. Diez L, Urbain L, Lejeune P, Detrain C. Emergency measures: Adaptive response to pathogen intrusion in the ant nest. Behavioural Processes. 2015;116:80-86.

42. Evans JD, Aronstein K, Chen YP, Hetru C, Imler JL, Jiang H, et al. Immune pathways and defence mechanisms in honey bees *Apis mellifera*. Insect Molecular Biology. 2006;15(5):645-656.

43. Gherman BI, Denner A, Bobis O, Dezmirean DS, Marghitas LA, Schluens H, et al. Pathogen-associated self-medication behavior in the honeybee *Apis mellifera*. Behavioral Ecology and Sociobiology. 2014;68(11):1777-1784.

44. Manson JS, Otterstatter MC, Thomson JD. Consumption of a nectar alkaloid reduces pathogen load in bumble bees. Oecologia. 2010;162(1):81-89.

45. Bos N, Sundström L, Fuchs S, Freitak D. Ants medicate to fight disease. Evolution. 2015;69(11):2979-2984.

46. Erler S, Moritz RF. Pharmacophagy and pharmacophory: mechanisms of self-medication and disease prevention in the honeybee colony (*Apis mellifera*). Apidologie. 2016;47(3):389-411.
